# Pharmacological evaluation of synthetic cannabinoids identified as constituents of spice

**DOI:** 10.1007/s11419-016-0320-2

**Published:** 2016-05-17

**Authors:** Cornelius Hess, Clara T. Schoeder, Thanigaimalai Pillaiyar, Burkhard Madea, Christa E. Müller

**Affiliations:** Department Forensic Toxicology, Institute of Forensic Medicine, University Hospital of Bonn, Stiftsplatz 12, 53111 Bonn, Germany; PharmaCenter Bonn, Pharmaceutical Institute, Pharmaceutical Chemistry I, University of Bonn, An der Immenburg 4, 53121 Bonn, Germany; Research Training Group 1873, University of Bonn, 53127 Bonn, Germany

**Keywords:** Synthetic cannabinoid, Structure-activity relationship, GPR18, GPR55, Indazoles, Indoles

## Abstract

In recent years, many synthetic cannabinoid (CB) receptor agonists have appeared on the market as constituents of herbal incense mixtures known as “spice”. Contrary to the declared use, they are perorally consumed as a replacement for marijuana to get “high”. In many cases, detailed information on the physicochemical and pharmacological properties of the synthetic compounds found in spice preparations is lacking. We have now evaluated a large series of heterocyclic compounds, 1,3-disubstituted indole and 2-azaindole derivatives known or assumed to be CB_1_ receptor agonists, many of which have previously been identified in forensic samples. The mainly observed structural variations to circumvent restriction by law were bioisosteric exchanges of functional groups in known CB_1_ agonists. We analyzed the structure-activity relationships of compounds at human CB_1_ and CB_2_ receptors based on affinities obtained in radioligand binding studies, and determined their efficacy in cAMP accumulation assays. Moreover, we investigated the activities of the compounds at the orphan G protein-coupled receptors GPR18 and GPR55 both of which are known to interact with cannabinoids. Most of the investigated compounds behaved as potent full agonists of CB_1_ and CB_2_ receptors with affinities in the low nanomolar to subnanomolar concentration range. Some compounds were moderately potent GPR55 antagonists, while none interacted with GPR18. Most derivatives were predicted to cross the blood–brain barrier as determined by bioinformatics tools. These data are useful for assessing synthetic cannabinoids and will be helpful for predicting pharmacological properties of novel compounds that appear on the illicit drug market.

**Electronic supplementary material:**

The online version of this article (doi:10.1007/s11419-016-0320-2) contains supplementary material, which is available to authorized users.

## Introduction

Cannabinoid (CB) receptors belong to the large family of rhodopsin-like class A G-protein-coupled receptors (GPCRs) [1]. The cannabinoid receptor 1 (CB_1_) was first described in 1993 as a major target for the natural product Δ^9^-tetrahydrocannabinol (Δ^9^-THC), the main psychoactive component of the herbal drug marijuana, derived from the plant *Cannabis sativa* [2]. The CB_1_ receptor is predominantly expressed in cells of the central nervous system, mediating the main psychoactive effects of Δ^9^-THC [3]. CB_1_ receptor activation is involved in analgesic and anxiety-related reactions, mediates appetite, and is peripherally involved in motor control and hypotension [4]. The CB_1_ receptor has long been discussed and tested as a drug target in metabolic diseases, relating to the fact that stimulation of CB_1_ receptors increases food intake, and its blockade reduces appetite [5, 6]. A second cannabinoid receptor (CB_2_) was subsequently discovered, which is predominantly expressed in the immune system, for example in the tonsils and spleen [7, 8], but has recently been described to be additionally expressed in the brain, mainly in microglia [9, 10]. CB_2_ receptors appear to be involved in inflammatory processes, and targeting this receptor may be a new approach to treat inflammatory diseases [11]. Both CB receptor subtypes display 44 % identity in amino acid sequences and are coupled to G_i/o_ proteins [2, 12]. Thus, activation of the receptors results in inhibition of adenylate cyclase, leading to reduced intracellular cAMP levels.

In recent decades, a broad range of potent synthetic CB receptor agonists and antagonists has been developed due to their potential for the treatment of various diseases including spasticity and neuropathic pain [13, 14]. Natural and synthetic CB_1_ agonists are widely abused due to their psychoactive, euphoric and analgesic effects, e.g., as ingredients of products commercialized as incense called “spice”. Due to this abuse, many of the synthetic CB agonists found in spice preparations are now on the list of controlled substances. However, the drug market is steadily flooded with new synthetic CB receptor agonists that are not yet subject to control by the authorities [15].

**Fig. 1:**
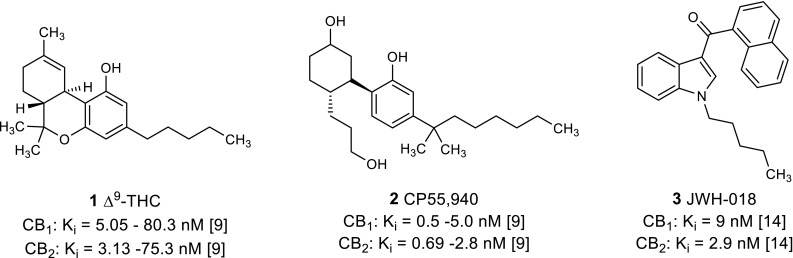
Structures and affinities of standard CB receptor agonists

The main classes of synthetic cannabinoid receptor agonists can be divided into the following major chemical classes: classical cannabinoids (dibenzopyrans, i.e., Δ^9^-THC, see Fig. 1), cyclohexyl-substituted phenols (i.e., CP55,940, see Fig. 1), naphthoylindoles, and benzoylindoles [16–18]. Based on these chemical structures—described and characterized in the scientific literature—novel derivatives have been commercialized via the Internet. Most of these new compounds consist of at least four structural components: 1. an indole or indazole core; 2. an ester, amide or ketone linker; 3. a ring consisting of a quinolinyl, naphthyl, adamantyl, tetramethylcyclopropyl or other moiety; 4. a hydrophobic “side chain” attached to the nitrogen atom of the indole or indazole ring system. They mainly display bioisosteric exchanges of substructures to circumvent legal prohibition. The pharmacological profiles of these compounds are often not known, and, therefore, forensic consequences for producers, traders and consumers cannot be anticipated since a scientific basis is lacking.

CB receptors are not the only targets of cannabinoids. Two “orphan” GPCRs—GPR18 and GPR55—have been reported to also interact with cannabinoids [11, 19]. “Orphan” receptors are characterized by the lacking of an endogenous ligand; therefore, their (patho-)physiology remains unclear. GPR18 was reported to be involved in microglial and endometrial migration processes [20, 21]. GPR55 is a receptor broadly expressed in the brain, partly co-expressed with both CB-receptors; its endogenous agonist was proposed to be lysophosphatidylinositol [22–24]. As the role of these poorly described orphan receptors remains largely enigmatic, new scaffolds for receptor ligands are required to further investigate the role of these receptors in human (patho-)physiology and to study their potential as drug targets.

In the present study, we investigated a series of compounds—collected by the Institute of Forensic Toxicology and Medicine, University of Bonn, based on the analysis of forensic samples—in radioligand binding assays for their interaction with both CB receptor subtypes, CB_1_ and CB_2_. Subsequently, the compounds were investigated for their functional properties in cAMP accumulation assays. Moreover, the potential of potent CB receptor agonists to cross the blood–brain barrier was estimated in silico. The compounds were additionally investigated for their ability to interact with the CB-like orphan receptors GPR18 and GPR55. The analysis of structure–activity relationships of the investigated compounds will help in predicting properties of novel derivatives.

## Materials and methods

### Materials

All compounds were obtained from Cayman Chemicals (Ann Arbor, MI, USA). According to the declaration by the manufacturer [liquid chromatography—tandem mass spectrometry (LC–MS/MS) data], the purity of all compounds was >95 %. We confirmed the purity in our laboratories by liquid chromatographic—mass spectrometry (LC–MS) measurements and found it to be >97 % for all compounds, except for two, RCS-8 (**34**; 92.3 %) and MAM-2201-4F-analog (**32**; 94.5 %). Compounds FUB-AKB48 (**18**) and A-834-735 (**46**) were synthesized in our laboratory at a multigram-scale and analyzed by LC–MS, ^1^H nuclear magnetic resonance (NMR) and ^13^C NMR spectroscopy (for details, see the supplementary material).

### Membrane preparations for CB receptor assays

Membranes of Chinese hamster ovary (CHO) cells recombinantly expressing the respective human CB receptor subtype, as described before [25], were prepared by scratching the cells off the previously frozen cell culture dishes in ice-cold hypotonic buffer (5 mM Tris-HCl, 2 mM EDTA, pH 7.4). The cell suspension was homogenized on ice for 1 min using an Ultra-Turrax (Ika, Higashiosaka, Japan) followed by further homogenization for 1 min with a dounce homogenizer, and subsequently spun down for 10 min at 4 °C and 1000*g*. The supernatant was centrifuged for 60 min at 48,000*g*. The obtained membrane pellets were resuspended and homogenized in the required amount of 50 mM Tris-HCl puffer, pH 7.4, to obtain a protein concentration at 5–7 mg/mL. Aliquots of the membrane preparation (1 mL each) were stored at −80 °C until being used [25].

### Radioligand binding assays at CB_1_ and CB_2_ receptors

Competition binding assays were performed using the CB agonist radioligand [^3^H](−)-cis-3-[2-hydroxy-4-(1,1-dimethylheptyl)phenyl]-trans-4-(3-hydroxypropyl)cyclohexanol (CP55,940, **4**, final concentration 0.1 nM; Perkin-Elmer Life Siences, Rodgan-Ingesheim, Germany) as previously described [26]. As a source for human CB_1_ and CB_2_ receptors, membrane preparations of the CHO cells stably expressing the respective receptor subtype were used (30 μg of protein/well for CB_1_ and 8 µg of protein/well for CB_2_ receptor preparations). Stock solutions of the test compound were prepared in dimethyl sulfoxide (DMSO). The final DMSO concentration in the assay was 2.5 %. After addition of 15 μL of the test compound in DMSO, 60 μL of [^3^H]CP55,940 solution in assay buffer, and 60 μL of membrane preparation to 465 μL of assay buffer [50 mM Tris, 3 mM MgCl_2_, 0.1 % bovine serum albumine (BSA), pH 7.4], the suspension was incubated for 2 h at room temperature. Total binding was determined by adding DMSO without a test compound. Nonspecific binding was determined in the presence of 10 μM of unlabeled CP55,940. Incubation was terminated by rapid filtration through a GF/C glass fibre filter (Perkin-Elmer, Boston, MA, USA) presoaked for 0.5 h with 0.3 % aq. polyethyleneimine solution, using a Brandel 96-channel cell harvester (Brandel, Gaithersburg, MD, USA). The filter was washed three times with ice-cold washing buffer (50 mM Tris, 0.1 % BSA, pH 7.4) and then dried for 1.5 h at 50 °C. Radioactivity on the filter was determined in a liquid scintillation counter (Topcount NXT, Packard/Perkin-Elmer, Boston, MA, USA) after 10 h of preincubation with 50 µl of scintillation cocktail (Multiscint 25, Perkin-Elmer). Data were obtained in three independent experiments, performed in duplicates. Data were analyzed using GraphPad Prism Version 4.02 (San Diego, CA, USA). For the calculation of *K*_*i*_ values, the Cheng-Prusoff equation and a *K*_*D*_ value of 2.4 nM ([^3^H]CP55,940 at CB_1_) and 0.7 nM ([^3^H]CP55,940 at CB_2_) were used [26].

### cAMP accumulation assays

Inhibition of adenylate cyclase activity was determined in CHO cells stably expressing the CB_1_ or the CB_2_ receptor subtype, respectively, using a competition binding assay for cAMP [25]. All details on the reagents and their origins were described in Ref. [25]. Cells were seeded into a 24-well plate at a density of 200,000 cells/well 24 h before performing the assays. After the incubation (see below), the cells were washed with Hank’s buffered saline solution (HBSS) consisting of NaCl (13 mM), HEPES (20 mM), glucose (5.5 mM), KCl (5.4 mM), NaHCO_3_ (4.2 mM), CaCl_2_·2 H_2_O (1.25 mM), MgSO_4_ (0.8 mM), MgCl_2_ (1 mM), KH_2_PO_4_ (0.44 mM), and Na_2_HPO_4_ (0.34 mM) dissolved in deionized, autoclaved water. After addition of 190 μL of HBSS per well, cells were incubated for 2 h at 37 °C. After this period of time, the phosphodiesterase inhibitor Ro-20-1724 [4-(3-butoxy-4-methoxybenzyl)-2-imidazolidinone, Sigma-Aldrich, St. Louis, MO, USA], at a final concentration of 40 μM dissolved in HBSS, test compound, and forskolin (final concentration: 10 μM, Sigma-Aldrich), all dissolved in HBSS containing 10 % DMSO, were added to each well. The final DMSO concentration was 1.9 %. The suspension was incubated for 10 min after the addition of Ro-20-1724, for 5 min after the addition of test compound, and for another 15 min after adding forskolin. cAMP accumulation was stopped by removing the supernatant from the cell suspension and subsequently lyzing the cells with 500 μL of hot lysis buffer (100 °C; 4 mM EDTA, 0.01 % Triton X-100). Aliquots of 50 μL of cell suspension were transferred to 2.5-mL tubes, into which 30 μL of [^3^H]cAMP (3 nM) and 40 μL of cAMP-binding protein (50 µg) were added, followed by 1 h of incubation at room temperature. The cAMP binding protein was obtained from bovine adrenal cortex as previously described [25]. Bound and free radioligands were separated by rapid filtration through a GF/B glass fibre filter (Perkin-Elmer). Radioactivity on the filter was determined in a liquid scintillation counter (TRICARB 2900TR, Packard/Perkin-Elmer) after 6 h of preincubation with 3 mL of scintillation cocktail (LumaSafeplus, Perkin-Elmer). Data were obtained from three independent experiments, performed in duplicates.

### β-Arrestin assays at GPR55 and GPR18

Recruitment of β-arrestin to the respective receptor was detected by using β-galactosidase enzyme fragment complementation technology (β-arrestin PathHunter™ assay, DiscoverX, Fremont, CA, USA) as previously described [27]. Data were obtained from three independent experiments, performed in duplicates. Data were analyzed using Graph Pad Prism Version 4.02 (San Diego, CA, USA).

### In-silico estimation of drug properties

Properties of compounds were predicted with the program Stardrop 5.5 (Optibrium, Cambridge, UK) using the ADME QSAR (quantitative structure-activity relationship) tool predicting the Lipinski rule of five, oral central nervous system (CNS) scoring profile and intravenous CNS scoring profile with standard conditions.

## Results and discussion

In this study, we investigated the CB receptor binding affinities and functional properties of three different classes of compounds structurally related to known CB receptor agonists. These compounds had been identified in “spice” preparations suspected to be commercialized for drug abuse (unpublished data).

### Binding affinities to CB_1_ and CB_2_ receptors

All compounds were investigated in radioligand binding experiments in CHO cell membrane preparations stably expressing the human CB_1_ or CB_2_ receptor using [^3^H]CP55,940 as a radioligand. *K*_*i*_ values are presented in Table 1. CB_1_ or CB_2_ selectivity of compounds was calculated based on the *K*_*i*_ values, and can be found in the electronic supplementary material (Table S1). All of the investigated compounds share a common core structure: (aza)indole. Three different types of linkers between the (aza)indole ring system and a bulky, lipohilic residue are observed: an amide, an ester or a shorter carbonyl linker. The most potent compounds for the CB_1_ receptor were found among the ester-linked subgroup [BB-22 (**27**), PB-22 (**24)** and 5F-PB-22 (**25**), NM-2201 (**19**)] with *K*_*i*_ values ranging from 0.217 to 0.468 nM. The only exception was the carbonyl-linked compound EAM-2201 (**31**), with a similarly low *K*_*i*_ value of 0.380 nM.

**Table 1:**
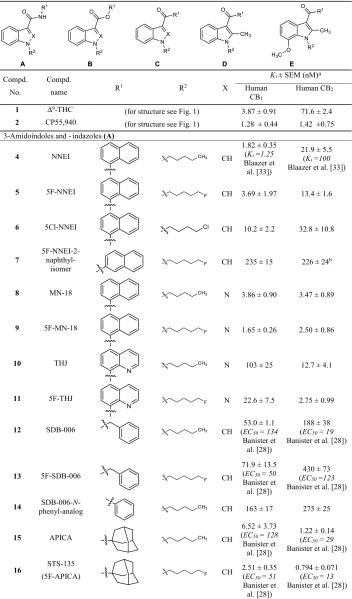

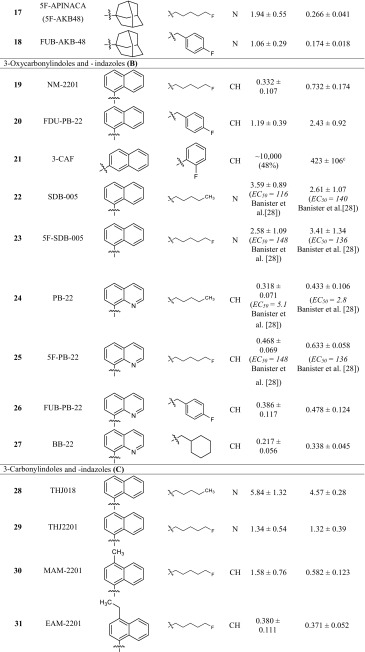

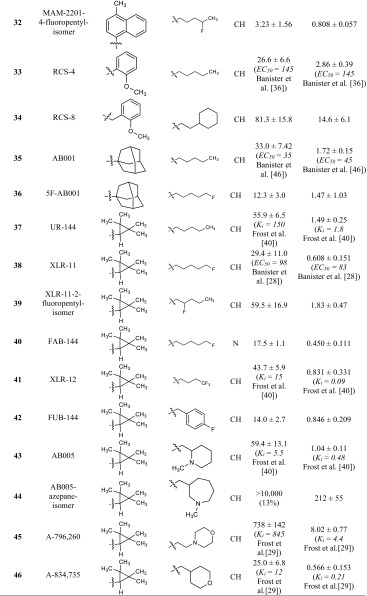

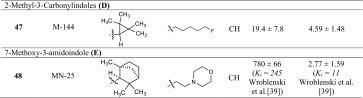
Affinities of investigated compounds at human CB_1_ and CB_2_ receptors

^a^Versus 0.1 nM [^3^H]CP55,940. For the experimental procedures see the section “Membrane preparations for CB receptor assays”. The *K*_*i*_ values are expressed as mean ± standard error of the mean (SEM) of three to five independent experiments. Literature data are given in brackets for comparison if available. EC_50_ values are from functional assays

^b^Maximal inhibition of radioligand binding: 80 % at 30 µM

^c^Maximal inhibition of radioligand binding: 73 %

In all three subgroups, compounds with typical bioisosteric exchanges are found. Three features of the molecule are varied: the *N1*-substituent, which was originally a pentyl moiety in the lead compounds of the JWH group [13]; in the current compounds, it is fluorinated or exchanged for a *para*-fluorobenzyl residue. The effect of fluorination on binding affinity was moderate: in the nine examples included in our study, binding affinity for the CB_1_ receptor was slightly enhanced for fluorinated compounds [compare MN-18 (**8**) and 5F-MN-18 (**9**); THJ (**10**) and 5F-THJ (**11**); APICA (**15**) and STS-135 (**16**); SDB-005 (**22**) and 5F-SDB-005 (**23**); THJ018 (**28**) and THJ2201 (**29**); and AB001 (**35**) and 5F-AB001 (**36**)] or slightly decreased [compare NNEI (**4**) and 5F-NNEI (**5**); SDB-006 (**12**) and 5F-SDB-006 (**13**); PB-22 (**24**) and 5F-PB-22 (**25**)]. Banister et al. [28] investigated the effects of fluorinated compounds and found that although the EC_50_ value of the investigated compounds were lower in vitro, this was not translated to higher in vivo potencies, leading to the assumption that pharmacokinetic effects play a role [28]. In their study, they investigated, amongst others, the pairs UR-144 (**37**) and XLR-11 (**38**), PB-22 (**24**) and 5F-PB-22 (**25**), and also APICA (**15**) and STS-135 (**16**). They performed membrane potential measurements using a fluorometric imaging plate reader (FLIPR) assay kit and determined slightly higher EC_50_ values for the compounds as compared to the radioligand binding data obtained in the present study. XLR-11-2-fluoropentyl-isomer (**39**), a derivative with a 2-fluoropentyl side chain, is the only compound in this series with a fluorine introduced at position 2 of the pentyl side chain. In comparison to the non-fluorinated analogue UR-144 (**37**), the affinity of **39** at the CB_1_ receptor was almost the same, but it was not as potent as XLR-11 (**38**), the 5-fluoinated derivative. MAM-2201-4-fluoropentyl-substituted isomer (**32**), showed also slightly higher *K*_*i*_ values than the 5-fluoropentyl derivative MAM-2201 (**30**). In this series, only one compound contains of a 5-chloro-substitution: 5Cl-NNEI (**6**), which displayed about five-fold lower affinity for the CB_1_ receptor than the unsubstituted derivative NNEI (**4**). Another bioisosteric replacement of the 5-fluoropentyl side chain is a *para*-fluorobenzyl residue. This variation is observed in four compounds of the present series [compare: 5F-AKB48 (**17**) and FUB-AKB48 (**18**); NM-2201 (**19**) and FDU-PB-22 (**20**); 5F-PB-22 (**25**) and FUB-PB-22 (**26**); XLR-11 (**38**) and FUB-144 (**42**)]. The affinity for both CB receptors was almost identical in three of the four pairs; only FDU-PB-22 (**20**) was not quite as potent as NM-2201 (**19**). Thus, a *para*-fluorobenzyl residue appears to be an optimal bioisosteric exchange for obtaining compounds with similarly high affinity as the 5-fluoropentyl-substituted parent compound.

Other side chains have been introduced at the indole nitrogen atom. Huffman et al. [13], who established alkylindoles as cannabinoid receptor ligands, already performed a comprehensive structure-activity relationship study introducing different side chains. They showed that a five-carbon side chain is preferred [13]. Thus, pentyl side chains and their bioisosteric analogs confer high potency and activity at the CB_1_ receptor. Whenever the size is decreased, affinity for the CB_1_ receptor is largely reduced. As this structural feature is crucial for high CB_1_ affinity, it had previously been modified to design CB_2_-selective compounds [29].

Another frequently observed variation is the replacement of the indole core by an indazole ring system. In the group of compounds with an amide linker (**A**), it could be observed that the affinity for the CB_1_ receptor was quite similar for indoles and indazoles, while the affinity for the CB_2_ receptor was slightly increased in indazole derivatives [compare NNEI (**4**) and MN-18 (**8**); 5F-NNEI (**5**) and 5F-MN-18 (**9**); STS-135 (**16**) and 5F-APINACA (**17**)]. In the group of compounds with an ester linkage (**B**), the indole derivative NM-2201 (**19**) showed lower *K*_*i*_ values at CB_1_ and CB_2_ receptors than the corresponding indazole derivative 5F-SDB-005 (**23**). In group **C** compounds containing a keto-group as a linker, XLR-11 (**38**) and its indazole analogue FAB-144 (**42**) displayed almost identical binding affinities. Thus, a variation of the heterocyclic core from indole to indazole is widely tolerated.

One other common feature of this group of compounds is the bulky lipophilic residue in position R^1^. Huffman et al. [16] introduced mainly naphthyl residues in that position. A variation of this structural element represents the introduction of a quinoline found in some compounds such as THJ (**10**) and PB-22 (**24**) [30]. In group **A** compounds with an amide linker, the introduction of a quinoline led to 14- and 27-fold higher *K*_*i*_ values at CB_1_ receptors, respectively [compare MN-18 (**8**) with THJ (**10**); and 5F-MN-18 (**9**) with 5F-THJ (**11**)], while the affinity for CB_2_ receptors remained unaltered in the low nanomolar range. In the ester-linked compounds (**B**), the quinoline-substituted analogue of NM-2201 (**19**), 5F-PB-22 (**25**), showed comparable affinities for both receptors. FUB-PB-22 (**26**) is a quinoline derivative with somewhat higher affinity at CB_1_ and CB_2_ receptors as compared to its analogue FDU-PB-22 (**20**). The most potent compound in this series of cannabinoid ligands, BB-22 (**27**)—sometimes referred to as QUCHIC—is also a quinoline derivative, which was first described in illicit drug material in 2013 in Japan [30]. This compound has a cyclohexylmethyl residue in position R^2^, which imitates the length of a pentyl chain that was previously described to be important for CB potency [13], and which was beneficial for CB_1_ receptor affinity also in a series of magnolol derivatives [31].

Compounds MAM-2201 (**30**) and EAM-2201 (**31**) display substitution of the naphthyl residue, containing a methyl (MAM-2201 (**30**) or an ethyl (EAM-2201 (**31**) group in position 4 of the naphthyl ring. EAM-2201 (**31**) was highly potent at the CB_1_ receptor with a *K*_*i*_ value of 0.380 nM without preference for any of the CB receptor subtypes. MAM-2201 (**30**), which had been described to cause severe toxicity in the cerebellum of rats [32], was found to be four times less potent at the CB_1_ receptor.

The only compound which is not linked in the 1-position of the naphthyl residue but is linked in the 2-position, 5F-NNEI-2-naphthyl-isomer (**7**), was a much weaker CB_1_ receptor ligand and also showed only partial inhibition of radioligand binding at the CB_2_ receptor. NNEI (**4**), which was first described by Blaazer et al. [33] in 2011, showed a *pK*_*i*_ value of 8.9 in their binding experiments at the CB_1_ receptor, which we have now confirmed. The authors also synthesized a non-fluorinated derivative of compound **7** (5F-NNEI-2-naphthyl-isomer) which displayed a lower *pK*_*i*_ value of 7.2 for the CB_1_ receptor. The same relation could be shown in the present study [compare 5F-NNEI (**5**) and 5F-NNEI-2-naphthyl-isomer (**7**)]; if the naphthyl residue is linked in 2-position to the amide, the affinity was decreased by about 100-fold.

Huffman et al. [13] investigated the effects of substituting the naphthyl ring by smaller aromatic residues, which reduced affinity to the CB_1_ receptor. This could also be observed for the benzyl-substituted compounds SDB-006 (**12**) and 5F-SDB-006 (**13**) investigated in the present study. They showed much lower affinity for both CB receptors as compared to the napthyl-substituted compounds with *K*_*i*_ values in the high nanomolar range. The phenyl-substituted derivative SDB-006-*N*-phenyl-analog (**14**) displayed even higher *K*_*i*_ values. In group **C** compounds, RCS-4 (**33**) and RCS-8 (**34**) also feature a phenyl or a benzyl residue. Wiley et al. [34] described that the substitution in the *ortho*-position is crucial for high affinity, which is realized in both compounds. RCS-8 (**34**), first described in 2012 in the USA [35], is benzyl-substituted in position 1 and has a cyclohexylethyl residue in position 2; it shows weaker affinity for both CB receptors than RCS-4. RCS-4 and isomers were investigated by Banister et al. [36] who found that RCS-4 (**33**) displayed EC_50_ values of 145 nM for CB_1_ and 46 nM for CB_2_. In the present study, RCS-4 (**33**) with *K*_*i*_ values of 26.6 nM for CB_1_ and 2.86 nM for CB_2_ displayed higher binding affinities.

The aromatic residue R^1^ may be replaced by a more bulky lipophilic group, namely an adamantyl or a tetramethylcyclopropyl residue. Comparing the naphthyl derivatives NNEI (**4**) and 5F-NNEI (**5**) with the adamantyl derivatives APICA (**15**) and STS-135 (**16**), it can be observed that CB_2_ affinity was increased. Also, the tetramethylcyclopropyl derivatives of group C displayed, independently of the side-chain variations, a CB_2_ preference. Compounds UR-144 (**37**), A-796,260 (**45**), A-834,735 (**46**) and XLR-12 (**41**) were first described by Frost et al. [29] in the search for selective CB_2_ agonists. We could confirm the reported *K*_*i*_ values, but only XLR-12 (**41**) displayed a 10-fold higher *K*_*i*_ value in our hands as compared to the literature data. From this group of compounds, some derivatives emerged on the illicit drug market, mainly in Sweden [37, 38]. FAB-144 (**40**), the indazole and 5-fluoropentyl analogue of UR-144 (**37**), showed slightly increased affinity for both CB receptors, and FUB-144 (**42**), the *para*-fluorobenzyl derivative displayed similar affinity. Also, compound M-144 (**47**), which is substituted in position 2 of the indole ring system with a methyl group, displayed a similar profile. AB-005 (**43**), a chimeric compound with the CB_2_ selectivity-increasing tetramethylcyclopropyl residue for R^1^ and *N*-methyl-2-piperidinylmethyl substitution as R^2^ which retains CB_1_ affinity, was first introduced by Frost et al. in 2010 [29]. A derivative with an azepane ring (**44**) appeared on the illicit drug market, but as we found, it displayed no affinity for the CB_1_ receptor at concentrations up to 10 µM. If it should exert any psychotropic effect, it would not be mediated via this receptor. At CB_2_ receptors, a moderate affinity was observed for **44**. A structurally related but more potent compound is MN-25 (**48**), which was introduced by Wrobelenski et al. [39]; it was reported to be abused in previous years [39].

In summary, almost all investigated compounds showed high affinity for CB receptors. Some compounds displayed *K*_*i*_ values in the subnanomolar range and, thus, are many times more potent than the psychoactive drug Δ^9^-THC.

### Functional properties of investigated compounds

**Fig. 2:**
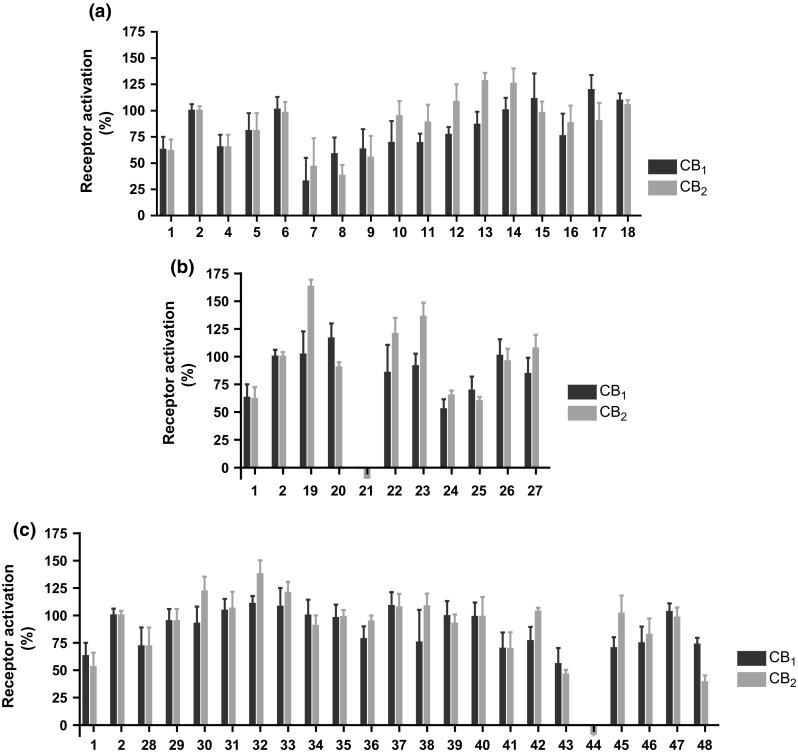
Functional properties of investigated compounds determined in cAMP accumulation assays, in the presence of forskolin (10 µM). Test concentration was 1 µM or 10 µM, depending on the determined *K*_*i*_ value. The selected concentration corresponds to the concentration at which a maximal effect was observed. All experiments were carried out three to five times, each in duplicate. **a** Compounds **4**–**18**; **b** compounds **19**–**27**; **c** compounds **28**–**24**. All results were normalized to maximal receptor activation by the full agonist CP55,940 (**2**)

To investigate the functional properties of the compounds, cAMP accumulation assays were performed. Both CB receptors are G_i_-coupled receptors, whose activation results in decreased cAMP levels in the cell. For comparison, the full agonist CP55,940 and the partial agonist Δ^9^-THC were investigated, and results were normalized to maximal receptor activation by the full agonist CP55,940 (see Fig. 2). Compounds were tested at a concentration where maximal binding was observed, either at 1 µM for the more potent compounds or at 10 µM for the less potent compounds.

In the utilized recombinant cell lines, Δ^9^-THC behaved as a partial agonist, at both CB_1_ and CB_2_ receptors, with 60–70 % activation as compared to the full CB_1_/CB_2_ agonist CP55,940 (**2**). Almost all compounds showed a high degree of activation of both receptor subtypes. Exceptions were 3-CAF (**21**) and AB-005 azepane isomer (**44**), which did not activate the CB receptors at all. As both compounds showed affinity for the CB_2_ receptor, they may be characterized as moderately potent CB_2_-selective antagonists. The only agonistic compounds with lower efficacy than Δ^9^-THC were NNEI-2-naphthyl isomer (**7**), MN-18 (**8**), XLR-12 (**41**) and AB005 (**43**). Most of the compounds had similar efficacies at both receptor subtypes; only 5F-APINACA (**17**) activated CB_1_ receptor more efficaciously than CB_2_.

**Fig. 3:**
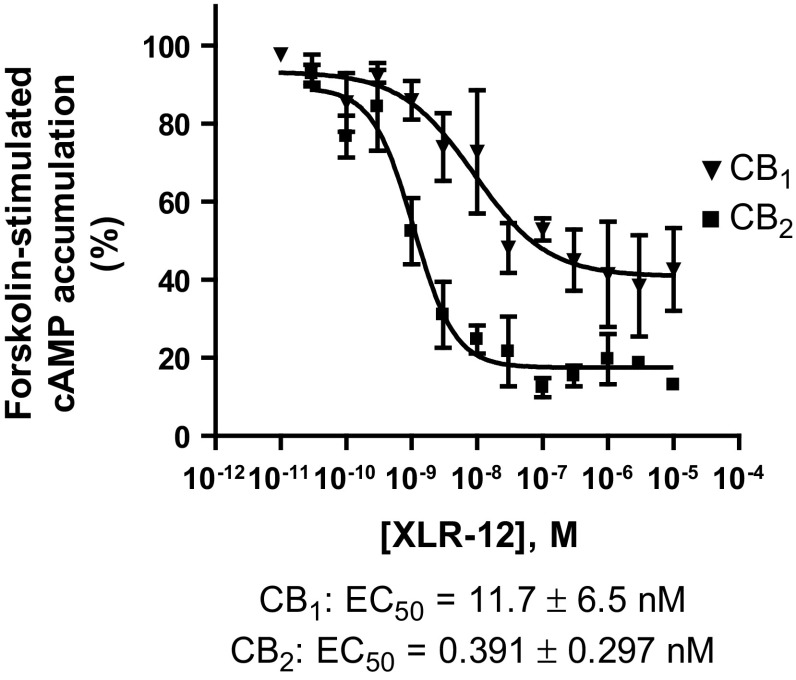
Concentration-dependent inhibition of cAMP accumulation by XLR-12 (**41**). All experiments were carried out three to five times, each in duplicate

For the CB_2_-selective derivative XLR-12 (**41**), full concentration response curves were recorded and EC_50_ values were determined (Fig. 3). It showed a 30-times lower EC_50_ value of 0.391 nM at the CB_2_ receptor than at the CB_1_ receptor; thus, the compound’s preference could also be observed in the functional assays.

*K*_*i*_ values measured in radioligand binding in many cases (Table 1) correlated quite well with reported and the EC_50_ values determined in cAMP accumulation assays (data not shown). CB_2_-selectivity of compound XLR-12 (**41**) could be confirmed, but in our hands it was lower (only 30-fold, Fig. 3) than previously reported one (167-fold) [40]. It should be emphasized that EC_50_ values depend on receptor expression levels while radioligand binding data are independent of receptor density or G protein expression. They directly reflect the affinity of compounds for the binding site on the receptors.

Compounds that activate the CB_1_ receptor to a comparable extent as Δ^9^-THC and that can cross the blood-brain barrier will likely cause similar psychotropic effects as Δ^9^-THC. Some compounds showed even higher efficacy than the full agonist CP55,940, including the very potent compounds EAM-2201 (**31**), NM-2201 (**19**) and BB-22 (**27**). Their toxicity may be much higher than that of Δ^9^-THC due to their high potency and full efficacy. PB-22 (**24**), a CB_1_/CB_2_ partial agonist with similar efficacy as Δ^9^-THC but with higher subnanomolar affinity (Table 1), had previously been reported to even cause lethal intoxications [41, 42].

### In silico prediction of drug properties

**Fig. 4:**
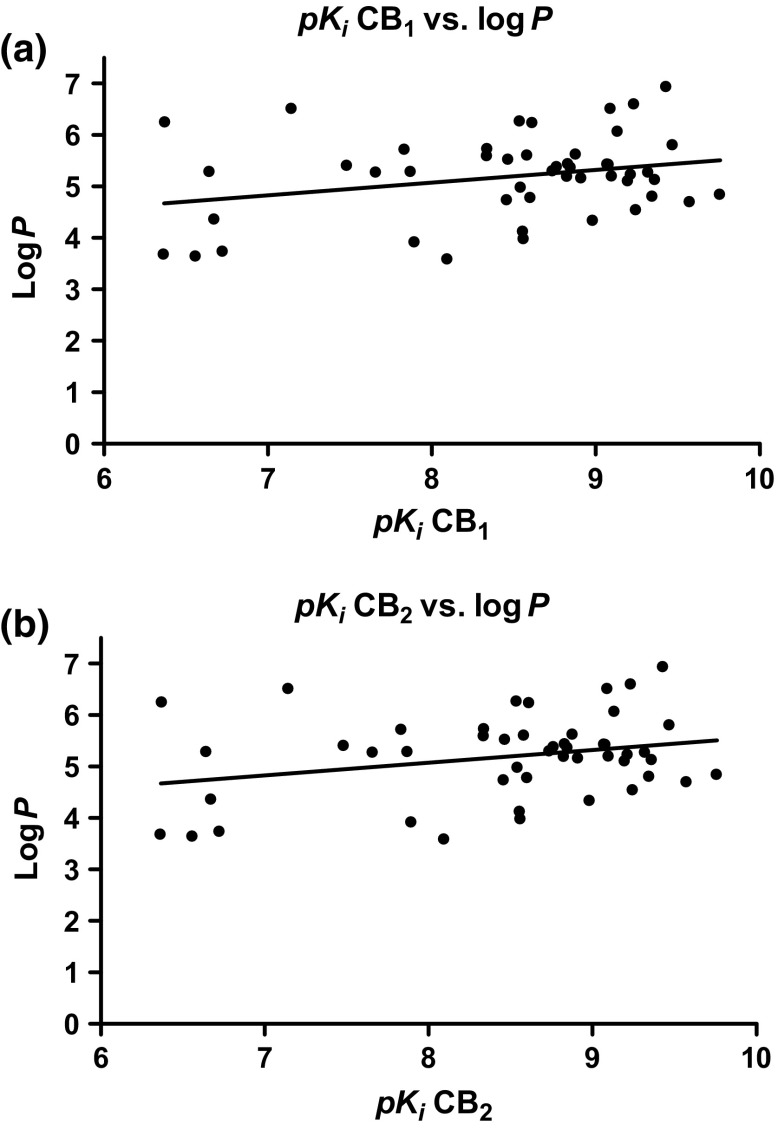
**a** Affinities of investigated compounds at the CB_1_ receptor plotted against log*P* values. **b** Affinities of investigated compounds at the CB_2_ receptor plotted against log*P* values

As a precondition to achieve psychoactive effects, brain penetration of the compounds is required. This property can be determined in animal studies. Alternatively, an in silico prediction based on established data sets can be used to gain an idea whether a set of compounds is able to cross the blood-brain barrier. For the investigated compounds, this was accomplished using the QSAR software Stardrop 5.4 (Optibrium). In Fig. 4, affinities of the investigated compounds were compared to their lipophilicity, which is one of the major determinants for crossing biomembranes. As can be observed, all compounds share a rather high log*P* value between 3 and 7. All highly potent compounds exceeded a log*P* of 4.5. The standard CB agonists displayed similarly high log*P* values of 6.50 (Δ^9^-THC), and 5.36 (CP55,940). The compounds’ potency is not directly correlated with their lipophilicity (see Fig. 4). Based on calculations to estimate lipophilicity (log*P*), topographical polar surface area and other parameters, a prediction whether compounds are able to cross the blood-brain barrier is made by the program. The compounds could thus be divided into two groups, blood-brain barrier-penetrant and non-penetrant compounds. THJ (**10**) and 5F-THJ (**11**), both of which are 3-(8-quinolinyl)amido-indazoles, were predicted not to cross the blood-brain barrier. Based on in silico predictions it is, however, likely that the majority of the investigated compounds has the ability to cross the blood-brain barrier.

### Effects on the orphan receptors GPR18 and GPR55

The orphan GPCRs GPR18 and GPR55 have been shown to be targeted by a range of cannabinoid receptor ligands [19, 29, 43]. Therefore, we investigated whether the investigated spice constituents also interact with these cannabinoid-related receptors (Table 2). None of the compounds was able to activate GPR18 or to inhibit GPR18 activation up to a concentration of 10 µM. At GPR55, some compounds were found to be moderate antagonists, namely, APICA (**15**) and STS-135 (**16**) with IC_50_ values of 3–5 µM, as well as several compounds from group **C**. EAM-2201 (**31**) was the most potent GPR55 antagonists of this series with an IC_50_ value of 1.86 µM. Interestingly, none of the ester compounds (**B**) showed any inhibitory effect, and most of the active compounds were tetramethylcyclopropyl-substituted derivatives containing the CB_2_-preferring structure. UR-144 (**37**), XLR-11-2-fluoropentyl-isomer (**39**) and XLR-12 (**41**), which feature a lipophilic aliphatic or fluoropentyl side chain, were more potent than A-769,260 (**45**) or A-834,735 (**46**) with a morpholine or tetrahydropyran substituent, respectively. A typical functional behavior of cannabinoids at GPR55 can also be observed here: although all of the identified GPR55 ligands were agonists at the CB receptors, they showed inhibitory effects at GPR55. The same had been demonstrated for the CB agonist CP55,940 (**2**) as well as other CB receptor agonists [27, 44]. On the other hand, CB_1_ receptor antagonists, such as rimonabant, are agonists of GPR55 [22, 27, 45]. Both receptors, CB_1_ and GPR55, were reported to be co-localized in the brain, and receptor heteromerization has been postulated [23, 24].

**Table 2:** Activities of test compounds in β-arrestin assays at human GPR55 and GPR18

	Compound	Human GPR55		Human GPR18	
		EC_50_ (µM; % activation)	IC_50_ (µM; % inhibition)	EC_50_ (µM; % activation)	IC_50_ (µM; % inhibition)
**1**	THC	**–**	14.2 [47]	4.61 [47]	–
**2**	CP55,940	–	1.61 [48]	–	5.99 [47]
(**A**) 3-Amidoindoles and -indazoles					
**4**	NNEI	>10 (26 %)	>10 (30 %)	>10 (42 %)	>10 (−15 %)
**5**	5F-NNEI	>10 (25 %)	>10 (−8 %)	>10 (−3 %)	>10 (−17 %)
**6**	5Cl-NNEI	>10 (28 %)	>10 (5 %)	>10 (1 %)	>10 (−13 %)
**7**	5F-NNEI-2-naphthyl-isomer	>10 (19 %)	>10 (20 %)	>10 (9 %)	>10 (5 %)
**8**	MN-18	>10 (27 %)	>10 (35 %)	>10 (2 %)	>10 (37 %)
**9**	5F-MN-18	>10 (38 %)	>10 (−5 %)	>10 (−26 %)	>10 (23 %)
**10**	THJ	>10 (11 %)	>10 (50 %)	>10 (4 %)	>10 (30 %)
**11**	5F-THJ	>10 (28 %)	>10 (10 %)	>10 (−17 %)	>10 (44 %)
**12**	SDB-006	>10 (−5 %)	>10 (36 %)	>10 (−9 %)	>10 (22 %)
**13**	5F-SDB-006	>10 (−3 %)	>10 (11 %)	>10 (13 %)	>10 (−24 %)
**14**	SDB-006-*N*-phenyl-analog	>10 (20 %)	>10 (1 %)	>10 (9 %)	>10 (−13 %)
**15**	APICA	>10 (11 %)	4.77 ± 1.69	>10 (8 %)	>10 (44 %)
**16**	STS-135 (5F-APICA)	>10 (1 %)	3.41 ± 0.47	>10 (−2 %)	>10 (30 %)
**18**	FUB-AKB-48	>10 (−11 %)	(83 %)	>10 (−27 %)	(69 %)
(**B**) 3-Oxycarbonylindoles and -indazoles					
**19**	NM-2201	>10 (17 %)	>10 (23 %)	>10 (−8 %)	>10 (32 %)
**20**	FDU-PB-22	>10 (11 %)	>10 (30 %)	>10 (30 %)	>10 (−4 %)
**21**	3-CAF	>10 (26 %)	>10 (41 %)	>10 (4 %)	>10 (10 %)
**22**	SDB-005	>10 (8 %)	>10 (23 %)	>10 (15 %)	>10 (24 %)
**23**	5F-SDB-005	>10 (21 %)	>10 (47 %)	>10 (21 %)	>10 (24 %)
**24**	PB-22	>10 (15 %)	>10 (-12 %)	>10 (−18 %)	>10 (26 %)
**25**	5F-PB-22	>10 (5 %)	>10 (-10 %)	>10 (−5 %)	>10 (−5 %)
**26**	FUB-PB-22	>10 (5 %)	>10 (24 %)	>10 (15 %)	>10 (8 %)
**27**	BB-22	>10 (9 %)	>10 (34 %)	>10 (2 %)	>10 (18 %)
(**C**) 3-Carbonylindoles and -indazoles					
**28**	THJ018	>10 (6 %)	8.20 ± 2.11	>10 (33 %)	>10 (−5 %)
**29**	THJ2201	>10 (−1 %)	>10 (47 %)	>10 (18 %)	>10 (21 %)
**31**	EAM-2201	>10 (−24 %)	1.86 ± 0.16	>10 (14 %)	>10 (4 %)
**32**	MAM-2201-4-fluoropentyl-isomer	>10 (−41 %)	3.07 ± 1.48	n.d.	n.d.
**35**	AB001	>10 (−14 %)	~10 (56 %)	>10 (−12 %)	~10 (62 %)
**36**	5F-AB001	>10 (19 %)	~10 (48 %)	>10 (−6 %)	~10 (18 %)
**37**	UR-144	>10 (−5 %)	6.70 ± 1.65	>10 (17 %)	>10 (14 %)
**39**	XLR-11-2-fluoropentyl-isomer	>10 (−8 %)	5.69 ± 1.95	>10 (24 %)	>10 (29 %)
**40**	FAB-144	>10 (5 %)	~10 (77 %)	>10 (2 %)	~10 (57 %)
**41**	XLR-12	>10 (−5 %)	4.56 ± 1.97	>10 (27 %)	>10 (13 %)
**42**	FUB-144	>10 (−3 %)	~10 (62 %)	>10 (−12 %)	~10 (74 %)
**43**	AB005	>10 (16 %)	>10 (39 %)	>10 (−38 %)	>10 (−2 %)
**44**	AB005-azepane-isomer	>10 (21 %)	>10 (18 %)	>10 (11 %)	>10 (−6 %)
**45**	A-796,260	>10 (−1 %)	14.3 ± 2.5^a^	>10 (20 %)	>10 (−10 %)
**46**	A-834,735	>10 (8 %)	6.88 ± 1.51^a^	>10 (6 %)	>10 (6 %)
(**D**) 2-Methyl-3-carbonylindole					
**47**	M-144	>10 (−5 %)	~10 (86 %)	>10 (−7 %)	~10 (67 %)
(**E**) 7-Methoxy-3-amidoindole					
**48**	MN-25	>10 (−8 %)	>10 (47 %)	>10 (−12 %)	>10 (30 %)

^a^Extrapolated values; a full curve could not be determined due to limited solubility

*n.d.* not determined

## Conclusions

In conclusion, we determined the binding affinity of a large number of synthetic compounds suspected to be constituents of spice herbal blends. Our results confirm that the majority of the investigated compounds behave as highly potent CB receptor ligands with affinities in the low nanomolar to subnanomolar concentration range. Furthermore, we could show that they behave as agonists with high efficacy. In an in silico approach, all except two derivatives were predicted to cross the blood-brain barrier, and, therefore, are likely to produce psychoactive effects. The main structural variations of the compounds represent typical bioisosteric exchanges altering the structure of the compounds to circumvent restriction by law, but to retain the intended psychoactive effects. Knowledge of classical medicinal chemistry provides, in these cases, powerful strategies to bypass controlled substances. In our study, we provide a comprehensive analysis of the structure-activity relationships of spice constituents including 27 compounds of previously unknown potency and efficacy. The obtained data were compared to those of established CB receptor ligands. In the future, this may help to predict pharmacological behaviour of novel compounds that appear on the illicit drug market.

The compounds were further investigated at the CB receptor-related orphan GPCRs GPR18 and GPR55. While no interaction with GPR18 was detected, some derivatives behaved as weak antagonists of GPR55. Because knowledge about these newly discovered orphan receptors is still very limited, our results contribute to a better understanding of their ligands’ structural requirements. Moreover, we have identified novel GPR55 antagonists that could be used as starting points for future optimization.

## Electronic supplementary material

Below is the link to the electronic supplementary material.

Supplementary material 1 (PDF 170 kb)
