# Supplementary material for: Pharmacological evaluation of synthetic cannabinoids identified as constituents of spice
Source: Forensic Toxicol. 2016 May 17;34:329–43. doi: 10.1007/s11419-016-0320-2 (PMC4929166; doi:10.1007/s11419-016-0320-2)
Supplement: Supplementary file 1 — Supplementary material 1 (PDF 170 kb) [file 11419_2016_320_MOESM1_ESM.pdf]

Electronic Supplementary Material

Pharmacological evaluation of synthetic cannabinoids identified as constituents of spice

Cornelius Hess [1], Clara T. Schoeder [2,3], Thanigaimalai Pillaiyar [2], Burkhard Madea [1], and Christa E. Müller [2,3]

[1] Department Forensic Toxicology, Institute of Forensic Medicine, University Hospital of Bonn, Stiftsplatz 12, D-53111 Bonn, Germany

[2] PharmaCenter Bonn, Pharmaceutical Institute, Pharmaceutical Chemistry I, University of Bonn, An der Immenburg 4, D-53121 Bonn, Germany

[3] Research Training Group 1873, University of Bonn, 53127 Bonn, Germany

*Address correspondence to:
Dr. Christa Müller
Pharmazeutisches Institut, Pharmazeutische Chemie I
An der Immenburg 4, D-53121 Bonn, Germany
Phone: +49-228-73-2301
Fax: +49-228-73-2567
E-Mail: christa.mueller@uni-bonn.de

C. Hess and C. T. Schoeder contributed equally to this study.

Table of Contents

**Page**	
S2	**Synthetic procedures and analytical data:** Synthesis of FUB-AKB48 (**18**) and A-834,735 **(46)**
S5	**Figure S1** Synthesis of FUB-AKB48 (**18**)
S5	**Figure S2** Synthesis of A-834,734 (**46**)
S7	**Figure S3**. Prediction of blood-brain barrier penetration
S8	**Table S1**. Selectivity index of selected compounds
S10	**References**

Synthetic procedures and analytical data

Synthesis of FUB-AKB48 (18)

Intermediate compounds **50** and **51** were synthesized according to literature procedures [49].

*Indazole-3-carboxylic acid ethyl ester (**49**)*

A cooled (~5 ^{o}C) solution of indazole-3-carboxylic acid (10 g) in ethanol (50 mL) was treated with concentrated H_2SO_4 solution (2 mL), warmed to room temperature and stirred for 10 min. The mixture was refluxed for 12 h. The reaction was poured onto ice-water (100 mL) and extracted with ethyl acetate (3x50 mL). The combined organic extracts were washed with saturated $NaHCO_3$ (2x50 mL), brine solution (2x50 mL), dried over $MgSO_4$ and the solvent was concentrated under reduce pressure to provide **49**. Yield 100%; brown solid; LC-MS (m/z): 191 $[M+H]^+$. Purity (LC-MS): 97%. The intermediate **49** was not further characterized by NMR analysis and directly used for the next step.

*1-N-(4-Fluorobenzyl)-1H-indazole-3-carboxylic acid ethyl ester (**50**)*

To a cooled (0 ^{o}C) solution of **49** (5 g, 34 mmol) in anhydrous tetrahydrofuran (THF, 50 mL) was added potassium tertiary butoxide (*t*BuOK, 4.19 g, 43 mmol, 1.1 equiv.) and stirred for 1 h. After this, a solution of 4-fluorobenzyl bromide (6.50 mL, 54 mmol, 1.6 equiv.) in THF (10 mL) was added very slowly (~5 min) *via* a syringe pump. The mixture was warmed to room temperature and stirred for 5 h. The mixture was poured into water and extracted with ethyl acetate (3x50 mL). The combined organic extracts were washed with brine (50 mL), dried over $MgSO_4$, and the solvent was removed under reduced pressure. The crude material was purified by flash-column chromatography to give **50** in 89% yield; white solid; ^{1}H-NMR (600 MHz, DMSO-d_6) δ 8.09 (dt, J = 8.2, 1.1 Hz, 1H), 7.86 (dt, J = 8.5, 0.9 Hz, 1H), 7.48 (dd, J = 8.3, 6.9 Hz, 1H), 7.42 – 7.26 (m, 3H), 7.23 – 6.83 (m, 2H), 5.78 (s, 2H), 4.39 (q, J = 7.1 Hz, 2H), 1.36 (t, J = 7.1 Hz, 3H); ^{13}C-NMR (151 MHz, DMSO) δ 14.373, 51.973, 60.573, 110.996, 115.567, 115.711, 121.486, 123.172, 123.454, 127.159, 129.763, 129.820, 132.926, 134.573, 140.472, 161.962. LC-MS (m/z): 299 $[M+H]^+$. Purity (LC-MS): 95%.

*1-N-(4-Fluorobenzyl)-1H-indazole-3-carboxylic acid (**51**)*

A cooled (~5 ^{o}C) solution of **50** (2 g) in ethanol (15 mL) was treated slowly with 2 N NaOH (3 mL), warmed to room temperature and stirred for 10 min. The mixture was refluxed for 12 h and cooled to room temperature. Ethanol was removed under reduced pressure and the residue was dissolved in water (10 mL) and acidified with 6 N HCl. The resulting solid **51** was filtered, washed with H_2O and dried at 40 ^{o}C overnight. Yield 98%; white solid: ^{1}H-NMR (600 MHz, DMSO-d_6) δ 13.07 (s, 1H), 8.09 (d, J = 8.2 Hz, 1H), 7.84 (d, J = 8.5 Hz, 1H), 7.46 (dd, J = 8.3, 6.9 Hz, 1H), 7.35 – 7.24 (m, 3H), 7.21 – 6.95 (m, 2H), 5.75 (s, 2H); ^{13}C-NMR (151 MHz, DMSO) δ 163.51, 162.64, 140.55, 135.48, 133.03, 130.04, 129.88, 129.82, 127.17, 126.99, 123.38, 123.16, 121.81, 121.48, 115.93, 115.71, 115.31, 110.87, 51.91. LC-MS (m/z): 271 $[M+H]^+$. Purity (LC-MS): 100%.

*N-((3s,5s,7s)-Adamantan-1-yl)-1-(4-fluorobenzyl)-1H-indazole-3-carboxamide FUB-AKB-48 **(18)***

To a solution of **51** (2.17 g, 8.0 mmol) in N,N'-dimethylformamide (DMF, 20 mL) was slowly added 1,1′-carbonyldiimidazole (CDI, 1.56 g, 9.6 mmol, 1.2 equiv.), and the mixture was stirred at room temperature for 45 min. After the mixture turned yellow, a solution of 1-adamantylamine (2.42g, 16 mmol, 2 equiv.) in DMF (10 mL) was added under an argon atmosphere. The mixture was heated to 120 ^{o}C with stirring for 7 h. The reaction was then cooled to ambient temperature, and poured onto ice-water (50 mL) and extracted with ethyl acetate (3x50 mL). The combined organic extracts were washed with brine (50 mL), dried over $MgSO_4$ and the solvent was removed under reduced pressure. The crude material was purified by flash-column chromatography to give FUB-AKB-48 (**18**). Yield 90%; White solid: ^{1}H-NMR (600 MHz, DMSO-d6) δ 8.15 (d, J = 8.2 Hz, 1H), 7.73 (d, J = 8.6 Hz, 1H), 7.42 (dd, J = 8.2, 6.8 Hz, 1H), 7.34 – 7.27 (m, 2H), 7.25 (t, J = 7.5 Hz, 1H), 7.20 – 7.09 (m, 3H), 5.71 (s, 3H), 2.10 (d, J = 2.9 Hz, 7H), 2.08 – 2.01 (m, 4H), 1.66 (d, J = 3.6 Hz, 7H); ^{13}C-NMR (151 MHz, DMSO) δ 161.13, 140.73, 138.32, 133.22, 129.62, 129.56, 127.01, 122.59, 122.43, 122.15, 115.69, 115.55, 110.58, 51.62, 51.36, 41.24, 40.11, 36.12, 29.06. LC-MS (m/z): 404 [M+H]+. Purity (LC-MS): 100%.

Procedure for the synthesis of compound A-834,735 (46)

The synthesis of compound A-834,735 (**46**) was performed by adapting a literature procedure [50].

*2,2,3,3-Tetramethylcyclopropanecarbonyl chloride (**52**)*

To a flask containing 2,2,3,3-tetramethylcyclopropane carboxylic acid (8.0 g, 56.0 mmol) was added thionyl chloride (60 mL). The resulting solution was refluxed at 75 ^{o}C for 3 h. The mixture was then cooled to room temperature and concentrated under reduced pressure. The residue was diluted three times with 20 mL of dichloromethane (CH_2Cl_2) and concentrated to remove any remaining thionyl chloride. This was repeated two additional times, and the material was used without further purification or characterization.

*1H-Indol-3-yl-(2,2,3,3-tetramethylcyclopropyl)methanone (**53**)*

To a solution of indole (5.5 g, 45 mmol) in 20 mL of anhydrous CH_2Cl_2 at room temperature was added a 1M solution of ethyl magnesium bromide in THF (52 mL, 52 mmol) dropwise *via a* syringe pump. After the addition was completed, the solution was stirred for 15 min and a 1M solution of $ZnCl_2$ in Et_2O (52 mL, 52 mmol) was subsequently added. The mixture was stirred for an additional 30 min, and then 2,2,3,3-tetramethylcyclopropanecarbonyl chloride (45 mmol) in 30 mL of CH_2Cl_2 was added via a dropping funnel under an argon atmosphere. The mixture was stirred for 6 h at room temperature and was then quenched with 50 mL of a saturated, aqueous solution of NH_4Cl. The mixture was poured into water and extracted with CH_2Cl_2 (3 x 50 mL). The combined organic extracts were washed with brine (50 mL), dried over $MgSO_4$, and the solvent was removed under reduced pressure. The crude material was purified by flash-column chromatography to give **52**. Yield 50%; brown solid; ^{1}H NMR (500 MHz, DMSO-d_6) δ 11.76 (s, 1H), 8.52 – 7.67 (m, 2H), 7.43 (dd, J = 7.7, 1.2 Hz, 1H), 7.20 – 7.14 (m, 1H), 7.14 – 7.06 (m, 1H), 2.21 (s, 1H), 1.25 (d, J = 1.5 Hz, 12H). ^{13}C-NMR (126

MHz, DMSO-*d6*) δ 16.57, 16.91, 23.38, 23.72, 30.74, 111.98, 119.60, 121.41, 121.66, 122.56, 125.43, 132.85, 136.59, 194.03; LC-MS (m/z): 242 $[M+H]^+$. Purity (LC-MS): 96%.

*Tetrahydro-2H-pyran-4-ylmethylmethanesulfonate (**53**)*

To a solution of tetrahydropyran-4-methanol (1.0 g, 8.6 mmol) in 20 mL of tetrahydrofuran (THF) at 0 °C was added triethylamine (4.10 mL, 29.4 mmol) followed by methanesulfonyl chloride (1.06 mL, 13.7 mmol). The mixture was stirred at 0 °C for 10 min and then the reaction mixture was stirred at room temperature for an additional 1.5 h. The reaction mixture was filtered through Celite and washed with THF (100 mL). The solution was concentrated under reduced pressure to give tetrahydro-2*H*-pyran-4-ylmethyl methanesulfonate, which was used without further purification and characterization.

*1-[(Tetrahydro-2H-pyran-4-yl)methyl]-1H-indol-3-yl}-(2,2,3,3-tetramethylcyclopropyl)-methanone (A-834,735, **46**)*

To a solution of **53** (1.2 g, 4.8 mmol) in 15 mL of DMF at 0 °C was added NaH (60% dispersion in mineral oil, 0.545 g, 24 mmol). This mixture was stirred at 0 °C for 10 min, warmed to room temperature, and allowed to stir for 30 min. The solution was again cooled to 0 °C and tetrahydro-2*H*-pyran-4-ylmethyl methanesulfonate (3.2 g, 16 mmol) in 15 mL of DMF was added via a syringe pump. After the addition was complete, the ice-bath was removed and the reaction mixture was warmed to 50 °C and stirred for 2 h. The mixture was cooled to ambient temperature, diluted with 50 mL of ethyl acetate and quenched with 10 mL of a saturated, aqueous solution of NH_4Cl. The mixture was poured into water and extracted with ethyl acetate (3 x 30 mL). The combined organic extracts were washed with brine (50 mL), dried with $MgSO_4$, and the solvent was removed under reduced pressure. The crude material was purified by flash-column chromatography to give **46**. Yield: 95%; brown solid; ^{1}H-NMR (600 MHz, DMSO-*d6*) δ 8.26 (s, 1H), 8.22 (dd, J = 7.8, 1.0 Hz, 1H), 7.59 (d, J = 8.2 Hz, 1H), 7.22 (dd, J = 8.3, 7.0, Hz, 1H), 7.19 – 7.10 (m, 1H), 4.14 (d, J = 7.2 Hz, 2H), 3.82 (dd, J = 11.7, 4.5 Hz, 2H), 3.21 (td, J = 11.7, 2.3 Hz, 2H), 2.17 (s, 1H), 2.10-2.08 (m, 1H), 1.39-1.36 (m, 2H), 1.36 – 1.27 (m, 2H), 1.25 (d, J = 8.6 Hz, 12H); ^{13}C-NMR (151 MHz, $CDCl_3$) δ 143.17, 140.96, 140.67, 138.91, 135.58, 131.04, 129.06, 126.82, 126.53, 125.17, 124.22, 122.98, 122.65, 114.77, 114.15, 71.208, 58.85, 39.79, 36.35, 23.81; LC-MS (m/z): 340 $[M+H]^+$. Purity (LC-MS): 95%.

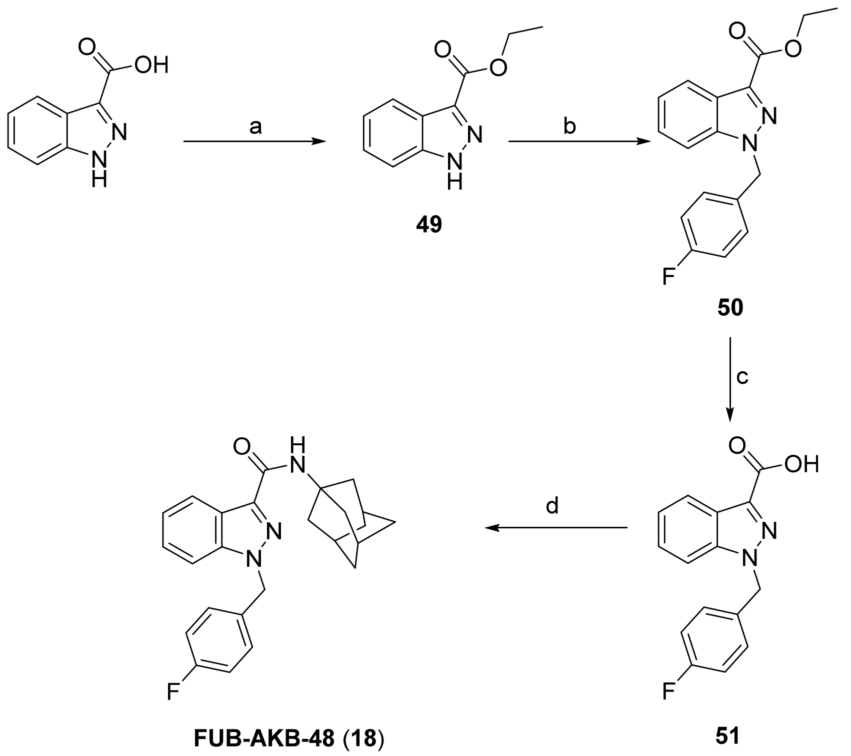

Figure S1: Synthesis of **18**. Reagents and condition: a) concentrated H_2SO_4, ethanol, reflux, 12 h, 100%; b) 4-fluorobenzyl bromide, *t*BuOK, THF, 5 h, 89%; c) 2 N aq. NaOH, ethanol, reflux, 1 h, 98%; d) 1-adamantylamine, CDI, DMF, 120 ^{o}C, 7 h, 90%.

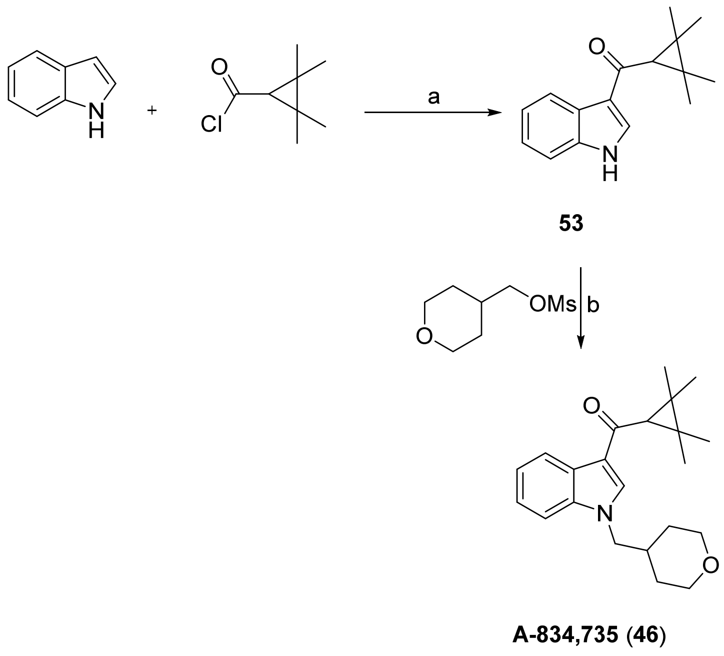

Figure S2: Synthesis of **46**. Reagents and condition: a) EtMgBr, $ZnCl_2$•Et_2O, CH_2Cl_2, 21 °C, 50%; b) NaH, DMF, 0 °C→50 °C, 2 h, 95%.

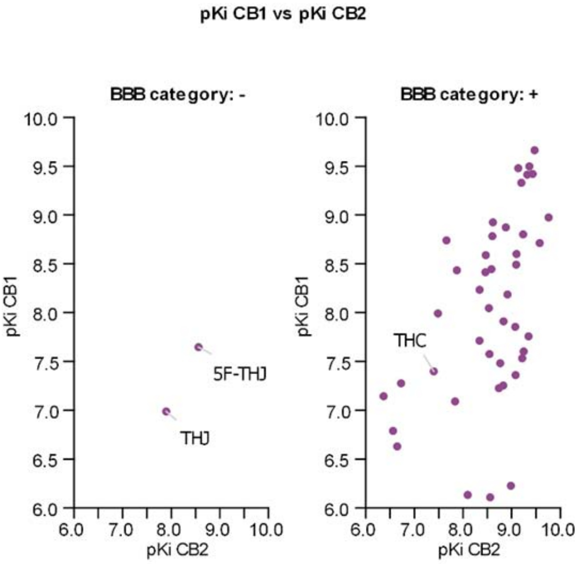

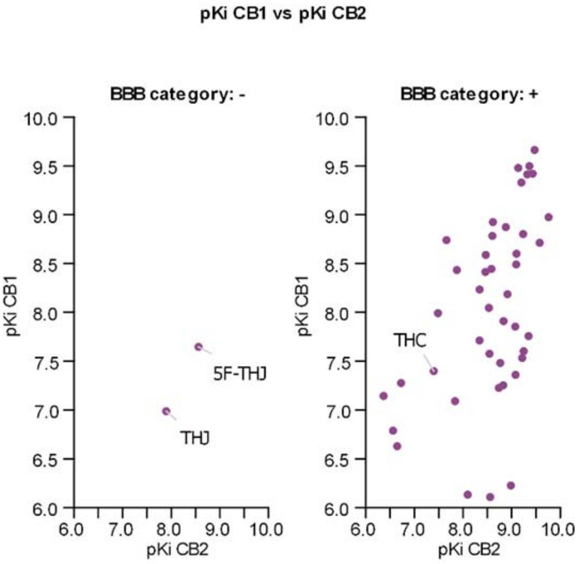

Figure S3. Blood-brain barrier category. Two groups of compounds were defined according to their predicted blood-brain barrier penetration. Stardrop 5.5 (Optibrium) defines scores based on established QSAR models. The prediction of the BBB category returns a binary prediction of penetration of the blood-brain barrier (+ : accuracy and specifity is 91%; - : accuracy and specificity is 83%).

Table S1. Selectivity index of selected compounds.

No.	Compd	CB_1 selectivity K_i CB_2/$K_i$$CB_1$	CB_2 selectivity K_i CB_1/$K_i$$CB_2$
**1**	THC	18.5	
**2**	CP55,940	1.1	
**4**	NNEI	12.0	
**5**	5F-NNEI	3.6	
**6**	5Cl-NNEI	3.2	
**7**	5F-NNEI-2-naphthyl-isomer	1.0	1.0
**8**	MN-18	0.9	1.1
**9**	5F-MN-18	1.5	
**10**	THJ		8.1
**11**	5F-THJ		8.2
**12**	SDB-006	3.5	
**13**	5F-SDB-006	6.0	
**14**	SDB-006-N-phenyl-analog	1.7	
**15**	APICA		5.3
**16**	STS-135 (5F-APICA)		3.2
**17**	5F-APINACA (5F-AKB48)		7.3
**18**	FUB-AKB-48		6.1
**19**	NM-2201	2.2	
**20**	FDU-PB-22	2.0	
**21**	3-CAF		23.6
**22**	SDB-005		1.4
**23**	5F-SDB-005	1.3	
**24**	PB-22	1.4	
**25**	5F-PB-22	1.4	
**26**	FUB-PB-22	1.2	

**27**	BB-22	1.6	
**28**	THJ018		1.3
**29**	THJ2201	1.0	1.0
**30**	MAM-2201		2.7
**31**	EAM-2201	1.0	1.0
**32**	MAM-2201-4-fluorpentyl-isomer		4.0
**33**	RCS-4		9.3
**34**	RCS-8		5.6
**35**	AB001		19.2
**36**	5F-AB001		8.4
**37**	UR-144		37.5
**38**	XLR-11		48.4
**39**	XLR-11-2-fluorpentyl-isomer		32.5
**40**	FAB-144		38.9
**41**	XLR-12		52.6
**42**	FUB-144		16.5
**43**	AB005		57.1
**44**	AB005-azepane-isomer		47.2
**45**	A-796,260		92.0
**46**	A-834,735		44.2
**47**	M-144		4.2
**48**	MN-25		282

References

(49) Banister SD, Moir M, Stuart J, Kevin RC, Wood KE, Longworth M, Wilkinson SM, Beinat C, Buchanan AS, Glass M, Connor M, McGregor IS, Kassiou M (2015) Pharmacology of Indole and Indazole Synthetic Cannabinoid Designer Drugs AB-FUBINACA, ADB-FUBINACA, AB-PINACA, ADB-PINACA, 5F-AB-PINACA, 5F-ADB-PINACA, ADBICA, and 5F-ADBICA. ACS Chem Neurosci. 6: 1546–59. doi: 10.1021/acschemneuro.5b00112

(50) Frost JM, Dart MJ, Tietje KR, Garrison TR, Grayson GK, Daza AV, El-Kouhen OF, Miller LN, Li L, Yao BB, Hsieh GC, Pai M, Zhu CZ, Chandran P, Meyer MD (2008) Indol-3-yl-tetramethylcyclopropyl ketones: effects of indole ring substitution on CB2 cannabinoid receptor activity. J Med Chem. 51: 1904–12. doi: 10.1021/jm7011613
